# Inquiry into the Influence of Chemistry on the Operation of Animal Bodies

**Published:** 1802-05

**Authors:** 


### *Inquiry into the Influence of Chemistry on the Operation of Animal Bodies.*

[ Continued from Numb. XXXVII. pp. 261—271. ]

THE objections we have advanced against the doctrines of the Chemical Sects are founded upon principles which this Sect is compelled to acknowledge; for its partizans admit that the origin of vitality cannot proceed from the change of combination in organic matter; but they maintain that when such an idea is received, it is alone to be considered as existing in the most exalted sphere of action in *transcendentalissimis*, that is, above the reach of our senses, and thus far beyond human comprehension; yet we ought to content ourselves with considering the cause of organic life, or of its appearances, applicable to beings already formed.

But if this doctrine were admitted, that the principles of whose nature we are ignorant must be considered as *non-existent*, all the power of Nature must be annihilated. We know as little of the principle or cause of attraction and repulsion as of vitality. The first causes of powers in Nature are concealed from us; and we know these as we know of vitality alone, by operations and appearances.

Thus this mode of reasoning fully demonstrates that the first principle of life is far above human comprehension, and that it may possibly remain an impossibility for us ever to penetrate into the *arcana* of Nature's operations, yet it in no wise demonstrates that a vital principle does not exist. On the contrary, that inconceivable uniformity of all the parts of organized nature with herself, and with each other, by which her incessant operations suggest the idea of some objective plan, necessarily leads us back to the acknowledgement of a separate principle, or a separate power, which is not operative in any particular matter simply, but throughout all nature, concerning the existence of which, although it be far beyond our comprehension, we draw the inference from those applicable appearances which are observable in living beings.

But it has been urged, vital power cannot be a distinct principle; for as the immortal *Kant* has justly remarked, the genuine idea of power is, *a priori*, a *compound* idea, which divides itself into the primitive ideas of *substance*, or *substantiality*, or *causation*; so that, whenever we contemplate two natural appearances in so close a union, that the one contains within itself the principle of the other, and they thus stand in the alternate relation of cause and effect, the connection of these two appearances with each other constitutes the idea of power, power. The idea of power is therefore a *subjective* idea, and by no means *objective*, as if *matter* and the *power* of matter were two diftinct ideas; for matter and power of matter are one and the fame. The metaphyfical idea of *matter* and *power* are not to be feparated; for *matter* is no other than *power* confidered in an *objective* point of view, and *power* no other than *matter* confidered in a *subjective* point of view. Thus organized bodies and organized powers are one and the fame, but contemplated in different points of view; and therefore a living power in the animal and vegetable kingdom is not to be confidered as a diftinct principle, but is to be inferred from the original and general power of matter.

However formidable this objection may appear, which is placed by the celebrated Dr. *Dieman* in all its force, and which is built upôn a fundamental axiom demonftrated by the philofophic *Kant*; yet it is our opinion that the *Kantifh* idea of power is not in the leaft inconfiftent with our conceptions of a diftinct principle of vitality, but that they perfectly agree with each other; for that organic bodies and organic powers are one and the fame, confidered in different lights; and that the *power* of operating, and *actual exertion*, ftand in reciprocal relation to each other, as caufe and effect, is with us indifputable: For we confider the organs as the medium or inftrument, and animal operations as the ultimate caufe of life. But this does not militate againft our conceptions of a feparate vital principle, as the firft and operative caufe of vitality; for we muft ftill inquire by what influence (to conform as much as poffible to the terms ufed by this diftinguifhed writer) is every thing in the objects of organized nature, rendered inftrumental in fuch a manner as to act upon each other in the moft intimate and regular manner, by virtue of which, the fame materials which compofe unorganized bodies are endowed with very different properties and powers when received into bodies that are organized; and that, which in the one ftate is merely a rude inactive mafs, is in living beings changed into the component parts of the firft organs, or into the conftituent parts of an irritable fibre or nerve poffeffing fenfation; whence this all-forming power, which produces an organ of fenfe from dead infenfible matter? "How comes it that in inanimate nature there prevails a certain uniformity of acting, and the changes which take place in objects are gradual and proceed from external caufes; while in animated nature all is activity, and harmonizes in certain ends; that there is an inceffant production and decay in beings, which from the moment of their exiftence to that of their diffolution, are carried on in one continued ftream of changes and alteralterations?" Can any one explain theſe ſingular and aſtoniſhing changes in their conſtitution, upon the principles of *attraction* and *repulsion*, the only powers known to be exiſtent in brute matter? If the appearances in organized nature are to be conſidered as ſo many ſeparate indications of theſe univerſal inherent powers, why is it that neither we, in any of our chemical experiments, nor Nature herſelf in her various operations, can, as we have already remarked, produce the ſmalleſt ſpark of vitality?

Our ſentiment is, that the vital principle is a principle diffuſed over univerſal nature, of itſelf formleſs, and alone operative where there is a capacity to receive its influence. It acts upon dead matter, which reacts according to its inherent properties. Both ſtand therefore in the mutual relation of cauſe and effect, and the attraction or relationſhip of theſe two to each other, forms our idea of living power.

We agree with Dr. Dieman in the remark, that "There is not a word more in uſe, to which various meanings are applied, than the word *power*. Power may be conſidered in three points of view, correſpondent with the idea of *poſſibility*, *agency*, and *neceſſity*. The firſt implies *inherent* power, the ſecond *operative* power, and the other the *powers* or the *laws of Nature*." This word may therefore be uſed to expreſs the *cauſe*, the *effects*, and the union of natural appearances as cauſe and effect. Thus, for example, the digeſtive faculty of the ſtomach we call a *power*, although the ſtomach may be deſtitute of food on which to act. Power in this caſe is the cauſe of digeſtion, and it conſiſts in an inactive *capability*. When this power is exerted in the act of digeſtion, and communicates peculiar properties to the food, this is an *effect* of its capacity; and it is now an *operative* power. When we ſay that the ſtomach of every living animal has the power of converting the food adapted to it, into a fluid containing ſpecific properties ſuited to the nature of the animal, no idea of neceſſary operation or law of Nature preſents itſelf. It is ſcarcely neceſſary to remark, that when we ſpeak of abſolute vital power, or of inherent power, in organized bodies, we conſider the term as expreſſive of the ſource or cauſe of *poſſibility*.

The laſt objection we ſhall mention, urged by the Chemical Sect is, that their ſentiments are founded on the teſtimony of our ſenſes, which in organized bodies preſent to our view nothing but *animal matter*; we muſt otherwiſe admit either an all-vivifying ſoul of the world, or give to each living body its particular ſoul.

This argument, inſtead of oppoſing our ſentiments, ſeems to confirm them; for all their chemical explanations proceed no no farther than the caufe of *vital appearances*, and are only applicable to living beings already formed. They are confined to the medium or *instrumental* caufe of vitality, and leave us in ignorance concerning the origin of this inftrumentality. Thus we feel ourfelves compelled to attribute to every organized body a primitive germ, by virtue of which each animal, each plant, is able to propagate its kind to infinity, but concerning the fpecific nature or origin of which we remain in the profoundeft ignorance. We may add, that even their fyftem demands an organic germ, to which the foreign fubftance may attach itfelf, in order to produce their animal cryftallization and re-production, and this muft be an organized part of an organized body, in order to have a fpecific affinity with the prepared matter; and confequently, a particular organic germ muft be fuppofed to exift for the production of bone, cartilage, mufcles, ligament, nerve, veffel, inteftine, &c. fince each diftinct or fpecific part can only be the effect of a fpecific cryftallization, or of a cryftallizing organ; and thus we muft afcribe to each of the numerous animal organs, its own particular germ, its own caufe of exiftence: Nay, according to this doctrine, the germs of each diftinct kind of exiftence in Nature muft be in readinefs, and, as it were, in a ftate of flumber, until it is awakened by its own natural ftimulant, and becomes the fpecific germ of fome particular organ. From hence it muft follow, that not only the germs of all the men who have peopled the earth, or that fhall hereafter exift, but alfo the germs of their refpective organs of the lungs; for example, liver, heart, ftomach, mufcles, nerves, of an infinite number of individuals, muft have exifted in miniature in our common parent *Eve!* A confequence too abfurd to be admitted!

We do not hefitate therefore to adopt with *Schelling*, *Sumarez*, and others, the fuppofition of a feparate principle as the caufe of vitality. It is this principle, by whofe energy the various conftituent parts of animal bodies are prevented from an union purely chemical, and preferves living beings from diffolution and putrefaction. It is this power which forms the various materials of the human body into an organized whole, and is to be confidered as the principal caufe of the individuality of every living being. For, in our opinion, the individuality, as characteriftic of every fpecies of living animals, proceed from, and is limited by this invifible principle. But this principle, although it is moft clofely united with the fpecific organization of animal bodies during life, is not, according to our ideas, a conftituent part of organization; nor is it fubject to any chemical changes, but by its uninterrupted influence fluence upon organized bodies supports life from the moment of their existence to that of their dissolution. It is thus the primary cause of the transmutation of rude and sluggish matter into vital and active organs, and the different aliments we use, are to be considered as unwrought materials, which are operated upon and organized by the living power, according to the constitution of different animals. It is the grand operator which changes the acorn into the oak, produces leaves and fruits from germs, and generates such a diversity of animals with the diversity of parts they possess from similar juices.

But although the living power is diffused over the whole of the animal frame, and every part possesses it during life, yet the different organs of the animal have specific powers superadded, by which the particular offices of different organs in animals are to be ascribed to the particular constitution and form of the organ. The liver, for example, the stomach, the tongue, and the eye, possess the common principle of vitality with the other parts of the body; but to the *liver* is superadded the particular power of changing the blood into gall; to the *stomach*, that of digesting different articles of food; the *tongue* enables us to judge of the sensible properties of bodies; and the *eye* communicates to the mind the impressions made by external objects on the optic nerves. These different powers become *operative* powers when they are excited to action by the stimulus of bodies adapted to their natures.

These specific powers, with their operation, are confounded by the Chemical Sect with its primary cause of vitality; whereas they are no other than the effects of the living principle, whose influence is indicated by the different organization of various parts; so that these various organic powers may be impeded, or even destroyed, while the living principle itself, which preserves them from dissolution and putrefaction, retains all its activity.

Though the stomach ceases to digest its food, it continues a living part of an organized being. The same is true of the liver, the tongue, and the eye; which in consequence of their particular construction may lose much of their peculiar powers, while they retain the common principle of life. The seeds of plants, eggs of some animals, and the inactivity of others during the winter season, and the fœtus in the womb, indicate the same fact.

The above remarks may make our ideas of life, and living power, more intelligible. We consider vitality as the result of an union of a peculiar principle with dead matter. This union producing a capability for various offices or functions, gives us the idea of different organic beings which we behold in the two two kingdoms of organized nature. *Living power*, on the other hand, is the production of the union of various ftimulants with the fum of organized powers. Particular living power, or rather fpecific exertions of living power, is the refult of the fpecific energetic power of a particular organ, and a particular ftimulant adapted to its nature; and is characterized by the peculiar conftruction of the part, and the nature of the ftimulant by which it is brought into action.

Of the various functions of animal life, *respiration* and *nutrition* are the moft neceffary to the human body; for the fufpenfion of thefe quickly becomes the caufe of death. They are both animo-chemical actions, that is, they are operations on which chemiftry has inconteftably a very great effect; but this is fo peculiarly modified by the vital power, that the refult is totally different from what is the natural product of chemical action on inanimate matter. This will appear evident by a minute attention to thefe two functions.

Refpiration is the firft and moft neceffary operation of the human body, the larger number of animal actions are more or lefs dependent upon it. It is by refpiration that animal bodies acquire the quantity of the acidifying principle neceffary to its exiftence. For the continuance of life it is not only an indifpenfible requifite, that the air fhould contain a certain portion of this principle, but that the fame fhould be either mechanically united with other parts of the atmofphere, or that the chemical union fhould be flight. For were the union to be more intimate, the vital power of the lungs would not be competent to the feparation of the neceffary quantity from the atmofphere. Refpiration becomes impeded, or totally fufpended. *Humboldt* adduces feveral inftances of this, where the air refpired contained from 0,26, to 0,27 parts of oxygen. He fuppofes that the principal caufe why carbonic acid gas is fo fatal to animal bodies, is to be afcribed to its intimate union with the oxygenic and the clofe affinity which fubfifts between them.

The two conftituent parts of the atmofphere are oxygen and azote, which are generally united in the proportions of 27 to 73. The reafon why the atmofphere is charged with fuch a preponderancy of irrefpirable air is, according to the opinion of Profeffor Reich, becaufe every indication of vitality is the refult of the mutual action of two oppofite principles; and becaufe that both conftitute the principle of life, though in different points of view. So that the *azote* is the *stimulating*, *sparkless*, and *positive* principle of vitality, and the oxygen is the *moderating*, *restrictive*, and *negative*.

We cannot fubfcribe to his fentiments; the laft pofition is pecularly objectionable. Azote and oxygen are deftitute of vitality vitality and fufceptibility of ftimulus, either in their pure unconnected ftate or in their numerous combinations with unorganized nature; and when the inftruments of refpiration are rendered motionlefs by death, neither azote nor oxygen are fufficient to reftore vitality to the body. This is a clear indication that thefe fubftances poffefs no vital power in themfelves, but that they communicate to the animal body the requifite degree of ftimulus: that the oxygen, in particular, is a kind of food adapted to the animal conftitution; and that after it has been acted upon by the lungs, and affimilated with the blood, it is to be confidered as one of the moft powerful caufes of preferving organized bodies in a pure and healthy ftate. The acidifying principle taken up by the lungs is, in fact, equally as deftitute of life as the food taken into the ftomach; and it is alone by the power of animated bodies, the particular living power of the inftruments both in refpiration and digeftion, that thefe different fubftances fhare in the vitality of the body, and are rendered capable either to improve or fupport the animal machine.

The principal caufe that the atmofphere does not confift of oxygen alone, but that it is mixed with fo large a portion of azote is, the ftimulating power of the former would be much too ftrong for the animal œconomy. For although it is the only part which is refpirable, and although, at firft, it renders refpiration quick and eafy, yet it would foon, by an accefs of ftimulus, excite inflammation in the lungs fatal to the fubject. This fact, which is proved by frequent experiments, is a demonftration that a preponderancy of azote is abfolutely requifite; and confequently, that the hypothefis of Profeffor Reich is deftitute of foundation.

But refpiration is not the only function upon which animal life depends; there are other extraneous fubftances, befides the atmofpheric air, to be acted upon and united with the vital organs. Thefe are introduced by the different articles of food. Nutrition cannot take place without the feparation of nutritive fubftances into their conftituent parts. Of confequence, digeftion is an animo-chemical procefs; and the different fecretions and excretions are alfo chemical operations, of a fecondary nature, contributing to the fame object.

We readily acknowledge that chemical powers have a great influence upon the procefs of nutrition. Nay, it appears to us highly probable that the grand object of digeftion is to feparate the *azote* from the other parts of nutritive bodies; and we are ready to grant, that the refult of fecretion and excretion confifts in fpecific feparations and combinations of the different animalized materials. But that the whole of this is chemical, is is a doctrine we cannot admit. Neither we, nor unorganized nature, have ever produced the leaſt appearance of animal fluids; and what an infinite number of organs are in continual exerciſe in order to change the food received into the body into animal juices! — A plain indication that nutrition is not a chemical proceſs ſimply, but that it is the reſult of an animo-chemical operation. We diſcover, it is true, by a chemical analyſis, that in moſt inſtances the ſame principles exiſt, but entirely changed in their properties, and in very different proportions. In ſhort, the more we contemplate the great diverſities, and the wonderful effects attendant upon nutrition, the more are we confirmed in our opinion, that although the effects produced are of a *chemical* nature, yet that the operative power is *animal*; otherways, it would be impoſſible that ſimilar and ſpecific fluids ſhould be produced from nutritious ſubſtances of ſuch different and contrary properties. Would it otherwiſe be poſſible that the requiſite quantity of phoſphorus ſhould exiſt in the body of a Gentoo, who lives entirely upon vegetables? How would it be poſſible that the ſame kind of food ſhould be adapted to the ſupport of different living beings and the peculiar organizations of each, and to the generation of their different fluids, if nutrition were a proceſs purely chemical.

What has been obſerved concerning theſe two leading functions in the animal œconomy, is alſo applicable to the others, which are more or leſs dependent upon them. Thus it appears to us that in the healthy ſtate of a living body, the organic power acts the firſt and principal part, to which chemiſtry is ſubſervient; but in its diſeaſed ſtate, particularly in diſeaſes of a certain claſs, chemical laws gain the aſcendancy, and act with ſuperior force.

This is the point of view in which we now conſider the ſubject, and ſuch are the reaſons which have induced us to relinquiſh the opinion we formerly ſupported, and embrace its oppoſite.* We aſcribe to organized Nature a diſtinct or poſitive principle, which does not prevent us from admitting a rational and moderate application of chemical powers to the phænomena obſervable in the animal and vegetable kingdoms.

---

* See Ontyd on Mortal Diſeaſes, London, 1798.

